# Integrating platelet-rich plasma therapy into nursing practice: a review of biological mechanisms and clinical applications

**DOI:** 10.3389/fbioe.2026.1711058

**Published:** 2026-02-13

**Authors:** Jing He, Darong Wang

**Affiliations:** 1 School of Nursing, The University of South China, Hengyang, Hunan, China; 2 Department of Nursing, The Central Hospital of Shaoyang, Shaoyang, Hunan, China

**Keywords:** musculoskeletal care, nursing protocols, platelet-rich plasma, regenerative nursing, wound healing

## Abstract

Platelet-rich plasma (PRP) therapy, an autologous biologic rich in growth factors, has emerged as a promising modality in regenerative medicine, with expanding relevance in nursing-led care. PRP promotes tissue regeneration, modulates inflammation, and enhances functional recovery in several conditions, including chronic wounds, musculoskeletal disorders, and aesthetic applications. Its minimally invasive nature and patient-specific approach align closely with holistic nursing models. This review synthesizes current evidence on the biological mechanisms underlying PRP activity, including platelet composition, growth factor signaling, and immunomodulation, and critically evaluates clinical outcomes relevant to nursing practice. Particular attention is given to nursing roles throughout the PRP treatment continuum, from patient selection and preparation to monitoring and long-term follow-up. Despite its clinical promise, PRP adoption in nursing practice faces barriers such as formulation variability, lack of standardized protocols, and limited nurse-led guidelines. We propose strategic directions for integrating PRP into evidence-based nursing frameworks, emphasizing digital tools, individualized care pathways, and interdisciplinary collaboration.

## 1 Introduction

Platelet-rich plasma (PRP) therapy has emerged as a promising modality in regenerative medicine. As its name suggests, PRP is autologous, highly biocompatible, and biologically active. It is obtained through centrifugation of a patient’s whole blood. It is a plasma fraction enriched in platelets at concentrations two-to eight-fold above baseline levels. PRP contains a complex mixture of growth factors, including cytokines and chemokines (Marck et al., 2019). Key biomolecules identified include platelet-derived growth factor (PDGF), transforming growth factor-beta (TGF-β), vascular endothelial growth factor (VEGF), and insulin-like growth factor-1 (IGF-1), all of which help in cell proliferation, extracellular matrix (ECM) remodeling, angiogenesis, and inflammation resolution (Montañez-Heredia et al., 2016). These molecular mechanisms underpin the capacity of PRP to promote favorable biological responses in acute and chronic tissue injury, positioning PRP as a biologically active adjunct or alternative to traditional therapeutic agents.

Over the last decade, PRP applications have expanded across numerous medical specialties, including orthopedics, sports medicine, dermatology, aesthetic medicine, and the management of chronic wounds. Additionally, its integration into nurse-led clinical activities has increased, particularly in cases characterized by impaired or delayed tissue healing, such as diabetic foot ulcers (DFUs) (Filova et al., 2021), venous leg ulcers (Senet et al., 2003), tendinopathies (Salini et al., 2015), and postoperative recovery (Ni et al., 2021). Evidence from randomized controlled trials and systematic reviews indicates that PRP may accelerate wound healing, reduce pain, and improve functional outcomes, especially when delivered within a structured, nurse-driven care setting (Moneib et al., 2018).

Despite a growing body of evidence supporting PRP therapy, its routine adoption in clinical practice remains limited. Several issues contribute to this limitation. PRP preparation methods are highly heterogeneous, varying from single-spin to double-spin centrifugation protocols. Moreover, different classifications exist (for example, leukocyte-rich and leukocyte-poor PRP). There is also no consensus regarding administration route, dosage, and treatment frequency. From a nursing implementation standpoint, although nursing considerations for patients receiving biologic therapies are demonstrated, monitoring parameters (for example, post-procedure monitoring, patient education, and adverse event monitoring) are generally not standardized.

As healthcare systems evolve toward multidisciplinary, patient-centered, and biologics-integrated systems of care, there is an urgent need to clarify, standardize, and expand the nursing role in PRP-based therapies. Accordingly, this review addresses existing knowledge gaps by integrating the biological foundations of PRP, evaluating its efficacy in areas relevant to nursing practice, and proposing a structured nursing workflow for PRP administration. Moreover, the review explores future directions for optimizing PRP implementation through digital health technologies, precision nursing approaches, and interprofessional collaboration.

## 2 Biological mechanisms of PRP

### 2.1 PRP composition and classification

PRP is a blood-derived biologic produced by centrifugation of autologous whole blood to concentrate platelets and lower the red blood cell fraction. The product is a PRP containing several bioactive components, including growth factors, cytokines, chemokines, and adhesive proteins.

PRP formulations are generally classified based on platelet concentration, leukocyte content, and fibrin architecture. This classification yields distinct categories such as leukocyte-rich PRP (LR-PRP), leukocyte-poor PRP (LP-PRP), and pure PRP (P-PRP), each exhibiting unique biological properties and therapeutic indications. For instance, LR-PRP may be advantageous for tendon or ligament healing but potentially harmful in intra-articular applications, as they can worsen synovial inflammation (Chen et al., 2020), depending on tissue context and inflammatory tolerance.

PRP preparation methods affect product quality and consistency. The most commonly used techniques are single-spin and double-spin centrifugation protocols, which differ in relative centrifugal force, spin duration, and temperature control (Charles et al., 2022). Platelet yield is affected by several factors, including white blood cell concentration, residual red blood cells, and fibrin content. Moreover, substantial variability exists among commercial PRP systems, contributing to heterogeneity in clinical trials and difficulties in direct comparison (Chahla et al., 2017; Dohan Ehrenfest et al., 2012).

It is important for nursing professionals who administer or participate in PRP protocol development to understand PRP classification and preparation variability. Such knowledge enables the selection of a suitable PRP formulation for specific pathologies and facilitates standardized documentation and outcome evaluation across care teams.

### 2.2 Growth factor function

The regenerative potential of PRP is due to the growth factors. These factors are stored within platelets’ alpha granules. Upon platelet activation, these growth factors are released, initiating a cascade of cellular and molecular events that drive tissue repair and remodeling (Kushida et al., 2014).

PDGF plays a role in the recruitment and proliferation of smooth muscle cells and fibroblasts (Liu T. et al., 2018; Wang et al., 2022). It also promotes ECM synthesis and granulation tissue formation (Kanno et al., 2019; Wang et al., 2017). TGF-β regulates collagen deposition and fibroblast activation and serves as an important modulator of fibrosis and inflammation (Kusuma et al., 2022; Ostapoff et al., 2014; Pan et al., 2021; Su et al., 2019). VEGF enhances neovascularization by stimulating endothelial cell proliferation and migration, resulting in better oxygen and nutrient supply to hypoxic and ischemic tissue sites (Hu et al., 2022; O'Connor et al., 2009; Wang et al., 2021).

IGF-1 (Liu et al., 2018b; Matsumura et al., 2017; Qiu et al., 2018) and hepatocyte growth factor (De Pasquale et al., 2018; Lee et al., 2022; Torres et al., 2019) support mesenchymal stem cell survival and differentiation, processes essential for tissue regeneration. Other factors, such as epidermal growth factor and fibroblast growth factor, contribute to epithelialization and keratinocyte proliferation during cutaneous wound healing (Jin et al., 2018; Manosalva et al., 2020; Rizzetto et al., 2022).

The therapeutic efficacy of growth factors depends on their concentration, timing of release, and spatial distribution. Excessive growth factor levels can impair healing, particularly in confined spaces such as joints, whereas insufficient concentrations may fail to produce considerable clinical effects (Sundman et al., 2011). Furthermore, growth factors exhibit tissue-specific responses, indicating a need for disease-oriented PRP formulations.

Nursing teams involved in wound care, orthopedic rehabilitation, and dermatologic interventions require adequate knowledge of growth factor biology to effectively educate patients, monitor expected tissue responses, and identify delayed or exaggerated healing patterns (Everts et al., 2020; Patel et al., 2023).

### 2.3 Intracellular signaling and immunomodulation

Following administration, PRP-derived growth factors bind specific receptors on target cells, triggering a series of intracellular events. The interaction of ligand and receptor activates PI3K/Akt (Lei et al., 2021), MAPK (Demy et al., 2024), and STAT3 (Ni et al., 2021; Kim et al., 2022) downstream signaling pathways. These pathways regulate cell survival, migration, proliferation, and angiogenesis. Together, these pathways provide a mechanistic basis for both regenerative effects and short-term local inflammatory responses observed after PRP administration.

In parallel, PRP exhibits considerable immunomodulatory effects. It enhances the production of anti-inflammatory cytokines, including interleukin-10 (IL-10) and IL-4 (Jayaram et al., 2023; Xu et al., 2025), thereby facilitating inflammation resolution and tissue homeostasis. Emerging evidence suggests that PRP may promote macrophage polarization toward the pro-healing M2 phenotype while suppressing the pro-inflammatory M1 phenotype (Heydari et al., 2024). In addition, microRNAs such as miR-192 and miRNA-26b-5p found in PRP-derived exosomes or platelet microparticles partially regulate MSC transition into osteogenic lineages (Karam et al., 2025; Rui et al., 2024).

PRP also promotes the formation of a three-dimensional fibrin matrix that functions as a temporary scaffold for cell migration and angiogenesis (Del Amo et al., 2020; Muiños-López et al., 2016). This matrix also stabilizes localized growth factor release and promotes clot formation at the site of injury.

In bone-related contexts, PRP enhances osteoblast precursor cells involved in bone regeneration and modulates BMP-2 and RUNX2 signaling to improve mineralization and bone formation. PRP has positive effects on BMP-2 and RUNX2 signaling (Cenni et al., 2009; Jiang et al., 2016; Salamanna et al., 2020; Wu et al., 2014).

An understanding of these molecular mechanisms is essential for researchers, clinicians, and nurses involved in treatment delivery, patient observation, and complication management. Such knowledge enables nurses to set realistic patient expectations and avoid unnecessary interventions by recognizing that transient pain or localized swelling may reflect a normal inflammatory response rather than treatment failure (Cullen and Gefen, 2023).

## 3 Clinical applications in nursing practice

PRP therapy has demonstrated therapeutic potential across multiple clinical contexts relevant to nursing practice, including musculoskeletal care, chronic wound management, rehabilitation, and aesthetic medicine. From a nursing perspective, PRP application extends beyond procedural support to encompass patient assessment, treatment coordination, monitoring, and education throughout the care continuum. This section summarizes current clinical evidence within these domains while highlighting the specific responsibilities and clinical decision-making roles of nursing professionals during PRP-based interventions.

### 3.1 Orthopedic nursing

PRP has gained prominence in orthopedic care for the management of degenerative joint diseases, tendinopathies, and post-surgical recovery. Evidence from randomized controlled trials suggests that PRP demonstrates favorable clinical effectiveness and safety in selected pain-related musculoskeletal conditions. Patients treated with PRP demonstrated better clinical outcomes at 6- and 12-month follow-up, with significantly lower pain recurrence and joint crepitus than the HA group (Hegab et al., 2015).

PRP also works well for tendinopathies such as rotator cuff tendinosis and lateral epicondylitis. Numerous studies report that PRP promotes resolution of inflammation, improves collagen fiber alignment, and shortens time to functional recovery (Moshiri et al., 2015; Zhou et al., 2015).

Nursing care in orthopedic PRP therapy involves a thorough pre-procedural assessment, including medication reconciliation—particularly regarding non-steroidal anti-inflammatory drugs (NSAIDs) and anticoagulants—screening for comorbidities such as diabetes and bleeding disorders, and patient education on post-procedure activity restrictions. During PRP administration, nurses must adhere to aseptic techniques and ensure proper patient positioning for comfort and safety. Post-procedurally, serial follow-up assessments using validated tools such as KOOS, WOMAC, or similar scoring systems are recommended (Mogoi et al., 2019; Wu et al., 2020). Nurses are also responsible for educating patients about the delayed onset of therapeutic effects and coordinating referrals to physiotherapy when appropriate. Although randomized controlled trials and meta-analyses generally support the superiority of PRP over hyaluronic acid or conservative management, heterogeneity in PRP formulations, injection protocols, and outcome measures limits direct comparison across studies. Awareness of these variations is essential for nurses when interpreting clinical responses, monitoring adverse reactions, and counseling patients regarding variability in treatment efficacy and recovery timelines.

### 3.2 Wound care nursing

Chronic wounds, including DFUs, pressure injuries, and venous leg ulcers, are critical challenges in nursing. PRP therapy improves wound closure by stimulating angiogenesis, re-epithelialization, and granulation tissue formation. In a clinical trial involving 70 patients with chronic cutaneous ulcers, topical application of autologous PRP gel for 4 weeks resulted in a 51% decrease in the median ulcer size (Mohammadi et al., 2017). Among patients with DFUs, PRP demonstrated superior outcomes compared with normal wound care. Further clinical trials have confirmed similar benefits in pressure ulcers and ischemic wounds (Fundiur et al., 2021; Putri et al., 2025). Interpretation should account for heterogeneity in wound etiology, severity, and delivery format (topical versus injection), which can influence healing trajectories.

From a nursing perspective, PRP-enhanced wound care requires advanced competencies in wound assessment, such as evaluation of wound stage, depth, exudate characteristics, and signs of infection. Nurse responsibilities include PRP application (via topical gel or intralesional injection), debriding when necessary, and applying moisture-retentive secondary dressings to the treated area.

Wound dimensions should be measured weekly during the treatment phase or according to institutional protocols, with accompanying photographic documentation (Mohammed et al., 2025). Adverse events, such as delayed epithelialization and signs of secondary infection, must also be closely monitored (Marques et al., 2023). Digital wound imaging systems and mobile documentation tools are increasingly adopted by nursing teams to standardize follow-up and enhance interdisciplinary communication. When PRP is incorporated into wound management, patient and caregiver education regarding offloading strategies, glycemic control, and adherence to dressing protocols becomes a critical component of nursing-led wound management. Despite consistent reports of accelerated healing and improved granulation, variability in wound etiology, PRP delivery methods, and follow-up duration affects evidence quality. Consequently, nursing-led wound assessment and standardized documentation are essential for translating heterogeneous evidence into safe, individualized, and reproducible clinical practice.

### 3.3 Sports rehabilitation nursing

PRP therapy has been widely studied for the management of soft tissue injuries involving muscle tears, ligament strains, and chronic overuse problems. It can help with lateral epicondylitis, plantar fasciitis, and Achilles tendinopathy management (Bilkis et al., 2025; Bucak et al., 2025; Huang et al., 2023). A double-blind, prospective, multicenter randomized controlled trial involving 230 patients with lateral epicondylitis demonstrated that PRP injection resulted in significantly lower rates of elbow tenderness at 24 weeks compared with controls (29.1% versus 54.0%; *p* = 0.009) and a higher overall treatment success rate (83.9% versus 68.3%; *p* = 0.037), supporting the clinical benefit of PRP over routine treatment in this population (Mishra et al., 2014).

In rehabilitation nursing, PRP therapy necessitates careful management of treatment timing and patient education. Nursing guidance typically progresses from initial rest and activity modification to gradual introduction of movement and eventual return to sport. Education on cryotherapy, limb elevation, compression, and safe resumption of training is integral to this process.

Psychological readiness and motivation are especially important among athletic populations. Nurses play a key role in managing expectations regarding tissue healing timelines. It may take 4–6 weeks after injection for maximal PRP effects to be achieved. Thus, reinforcement of adherence, pain monitoring, and tele-rehabilitation support can enhance adherence and satisfaction.

Injury surveillance also falls within the nursing scope of practice. Nurses should monitor for post-injection flare reactions, hematoma formation, and signs of compartment syndrome, particularly following high-impact injuries or deep intramuscular lesions (Bazezew et al., 2024). Close communication with sports physicians and physiotherapists is essential to optimize PRP administration timing and integrate treatment within comprehensive rehabilitation plans (Robertson et al., 2020). Accordingly, while PRP has demonstrated benefit in selected soft tissue injuries, clinical outcomes remain variable across injury types and athletic populations. Rehabilitation nurses play a key role in aligning patient expectations with the delayed biological effects of PRP and integrating treatment within structured, phased return-to-activity programs.

### 3.4 Aesthetic and reconstructive nursing

Using PRP in dermatology and aesthetic medicine is on the rise owing to its excellent safety profile, autologous origin, and dermal regeneration properties. Common clinical indications include facial rejuvenation, atrophic acne scarring, alopecia, and improvement of skin texture and tone (Ahramiyanpour et al., 2025; Alam et al., 2018; Huang et al., 2025; Somasekharan et al., 2020). A retrospective study involving 45 patients with androgenetic alopecia demonstrated that treatment with PRP alone or PRP combined with 5% minoxidil resulted in significant increases in hair count, hair density, terminal hair counts, and the anagen-to-telogen ratio (*p* < 0.05 in both groups). The combined therapy group also exhibited reduced vellus hair count and density compared with baseline (*p* < 0.05) (Koç Babayiğit et al., 2025). Another retrospective analysis of 56 patients treated with PRP for alopecia discovered a strong relationship between patient-reported improvement and hair density (*p* = 0.0006), but not hair diameter (*p* = 0.2688). The group that improved the most demonstrated the highest density gain (+18.9 hairs/cm^2^), whereas the group with the poorest response demonstrated a decrease in hair density (−19.7 hairs/cm^2^; *p* = 0.0012). These findings indicate that PRP efficacy in alopecia is best reflected by changes in hair density, which highlights the importance of integrating objective trichromatic measurements with subjective assessments during clinical evaluation (Brinks et al., 2025).

PRP has demonstrated promising regenerative and antimicrobial efficacy in burn wound management, making it a valuable adjunct in nursing-led wound care protocols. A meta-analysis of 9 randomized controlled trials including 413 patients reported that PRP therapy significantly accelerated wound closure, reducing healing time by a mean of 6.68 days. Moreover, PRP treatment was associated with a lower risk of wound infection (odds ratio [OR]: 0.18, 95% confidence interval [CI]: 0.04–0.88). Furthermore, PRP significantly reduced the number of dressing changes (mean difference [MD] −14.50; 95% CI: −16.45 to −12.55) and increased the proportion of healed wound area (MD +6.82%; 95% CI: 2.58–11.06). No significant differences were observed in pain score and graft take between PRP-treated and control groups (Yi et al., 2025). These findings indicate that PRP can accelerate the wound healing process, reduce the nursing workload associated with dressing changes, and reduce infection risk, thereby supporting its integration into evidence-based burn care.

Aesthetic PRP nursing care requires thorough patient assessment, appropriate skin preparation, including antiseptic cleansing and anesthetic application, and administration via microneedling or mesotherapy. Post-procedure nursing care focuses on pain management, infection prevention, and patient education regarding expected outcomes such as transient erythema, edema, or delayed pigmentary changes. Nurses also play a pivotal role in managing patient expectations, emphasizing that visible improvement in skin quality or appearance may not be immediate, with results typically observed within 2–4 weeks and optimal benefits achieved after multiple sessions (Asubiaro and Avajah, 2024; Hassan et al., 2025).

Aesthetic follow-up protocols generally include the evaluation of patient satisfaction, documentation of adverse events, and serial photographic assessments. Patient satisfaction is a key outcome in cosmetic nursing; thus, nurse-led communication, empathy, and responsiveness to patients’ aesthetic concerns are essential components of high-quality care. Clinical responses to PRP in aesthetic and reconstructive applications are influenced by treatment frequency, delivery technique, and patient-specific factors, resulting in variable outcome measures across studies. Therefore, nursing assessment, patient education, and longitudinal follow-up are essential to ensuring realistic expectations and consistent evaluation of therapeutic benefit.

## 4 Standardized nursing protocol for PRP therapy

To ensure the safe, effective, and reproducible application of PRP therapy, standardized nursing workflows are essential. From a nursing process perspective, PRP care encompasses structured assessment, preparation, procedural support, post-treatment monitoring, and comprehensive documentation. Establishing evidence-informed protocols facilitates consistency in clinical practice, reduces preventable adverse events, supports patient education, and enhances interdisciplinary coordination across care settings.

### 4.1 Pre-treatment nursing assessment and patient preparation

A comprehensive pre-treatment nursing assessment is fundamental to aligning PRP therapy with individual patient risk profiles, clinical indications, and expected outcomes. A detailed medical history should include screening for hematologic diseases (for example, thrombocytopenia), autoimmune diseases, active infection, malignancy, and recent steroid use. Particular attention must be paid to concurrent medications, as antiplatelet agents and NSAIDs may impair platelet activation (Hoefer et al., 2015; Nilsson et al., 2010; Panova-Noeva et al., 2020). These findings inform readiness and risk stratification, guiding whether PRP proceeds and what formulation- and indication-specific precautions are required.

Baseline laboratory tests may include a complete blood count to confirm adequate platelet levels (typically >150,000/μL). In wound care populations, CRP or glycemic markers may be assessed to guide risk stratification and treatment planning. Imaging, such as musculoskeletal ultrasound or X-ray, may be used to characterize pathology and optimize anatomical targeting of PRP injections.

Patient education is a key aspect of nursing-led preparation. Nurses should clarify that PRP is autologous, explain the rationale for using PRP, and mention the expected timeline of response (commonly 2–24 weeks) and the common side effects, which include temporary pain, swelling, or stiffness (Le et al., 2018). Patients should be advised to discontinue non-selective NSAIDs for at least 5–7 days before PRP administration, as these agents may inhibit platelet activation and alter growth factor release. When anti-inflammatory therapy is required, COX-2–selective NSAIDs may be used because of their lesser impact on platelet function (Evans et al., 2021; Kao et al., 2022). It is generally recommended to avoid non-selective NSAIDs 1–2 weeks following PRP treatment to ensure optimal activity (Hall et al., 2009). Written informed consent must be obtained following counseling. Pre-procedure fasting or hydration requirements must be followed per institutional standards.

Nursing personnel are also responsible for ensuring the availability of procedural equipment (for example, PRP centrifugation kits, sterile syringes, and activators), maintaining sterilization of the procedure room, and coordinating with cross-disciplinary teams before the procedure day.

### 4.2 Intra-procedural nursing support and aseptic technique

During PRP preparation and administration, nursing responsibilities center on procedural safety, infection control, real-time patient monitoring, and accurate documentation to ensure procedural fidelity and patient comfort. PRP preparation begins with atraumatic venipuncture using a sterile, closed blood collection system, followed by prompt processing to preserve platelet viability. Most validated double-spin protocols recommend an initial soft spin at 100–300 *g* for 5–10 min to separate plasma from red blood cells, followed by a hard spin at 400–700 *g* for 10–17 min to concentrate platelets while minimizing premature activation and hemolysis. Some studies report that higher centrifugal forces (800–2,000 *g*) during the second spin may increase yield but also raise the risk of platelet activation and may alter growth factor release profiles. Selection of centrifugation parameters should align with the PRP formulation and clinical indication (Dhurat and Sukesh, 2014; Perez et al., 2014). For nursing documentation, recording device-specific parameters supports traceability and helps distinguish protocol-driven steps from locally adapted practice.

Aseptic conditions must be strictly maintained throughout PRP preparation and administration. Nurses are responsible for establishing a sterile field and disinfecting injection sites with appropriate antiseptic solutions, while all staff must adhere to personal protective equipment standards. When exogenous activation agents (for example, calcium chloride or thrombin) are used, activation should be performed immediately before administration to preserve growth factor viability.

Real-time imaging guidance (for example, ultrasound or fluoroscopy) is strongly recommended for deep tissue or intra-acetabular injections to facilitate accurate probe placement, gel application, and interpretation (Huang et al., 2022; Sebbagh et al., 2023). Patients should be offered pain management measures such as topical lidocaine, ethyl chloride spray, or pre-treatment cryotherapy to enhance comfort. Continuous monitoring for vasovagal reactions or anxiety is required throughout the procedure.

Documentation at this stage should include platelet concentration (if available), the specific PRP type (for example, LR-PRP or LP-PRP), injection site, administered volume, and any immediate adverse events such as pain, dizziness, or bleeding (Mogoi et al., 2019).

### 4.3 Post-treatment follow-up, evaluation, and documentation

Post-treatment nursing care emphasizes systematic follow-up, outcome evaluation, complication surveillance, and patient education to support functional recovery and long-term treatment effectiveness. Typical follow-up schedules include assessments for 1–2 weeks, 4–6 weeks, and 12 weeks post-injection, although timing may vary based on the clinical indication (for example, orthopedic, dermatologic, or wound-related applications). Follow-up timing should be aligned with indication-specific outcomes (e.g., pain/function versus wound closure trajectory) and anticipated onset of PRP effects.

Validated assessment tools, such as the Visual Analog Scale, WOMAC (for osteoarthritis), or the Disabilities of the Arm, Shoulder, and Hand score, should be used to quantify changes in pain and function (Hudak et al., 1996; Huskisson, 1974). Wound healing outcomes should be evaluated using the Bates–Jensen Wound Assessment Tool or the Pressure Ulcer Scale for Healing; also include photograph documentation and use electronic wound mapping systems (Stotts et al., 2001).

Localized discomfort, erythema, stiffness, and mild swelling commonly observed after PRP injection typically resolve spontaneously within 48–72 h. Patients are generally advised to avoid NSAIDs, particularly non-selective cyclooxygenase inhibitors, for 5–7 days to optimize platelet function and growth factor release (Eymard et al., 2021). Recommended analgesic strategies include acetaminophen or cryotherapy. Patients should be instructed to promptly seek medical evaluation if concerning symptoms arise, such as fever, purulent discharge, or neurological symptoms.

Patient-reported outcomes, satisfaction scores, and rehabilitation adherence should be integrated into documentation systems. Digital health platforms, including secure applications or patient portals, can facilitate virtual follow-ups, symptom diaries, and automated reminders.

Nurses also play a critical role in reinforcing post-procedure activity guidelines, such as joint unloading, wound offloading, and sun avoidance following facial PRP treatments, and in arranging additional sessions when multiple treatments are planned. Patients should receive written post-discharge instructions and direct nursing contact information for follow-up inquiries.

## 5 Challenges and future directions

Despite growing interest in PRP therapy, several challenges continue to limit its integration into nursing practice. A major challenge is the heterogeneity of existing evidence, including variability in PRP composition (for example, leukocyte-rich versus leukocyte-poor formulations), preparation techniques, dosing regimens, and clinical endpoints. These variabilities complicate evidence synthesis and limit the development of universally applicable nursing protocols. Consequently, nurses must interpret PRP-related outcomes within the context of formulation-specific effects and individual patient responses.

Although PRP therapy is generally considered safe and minimally invasive, it is not devoid of complications. Local pain, swelling, and inflammatory flares are reported more frequently following LR-PRP administration. A systematic review and meta-analysis (n = 32 studies) demonstrated that LP-PRP and LR-PRP yield clinically meaningful improvements in pain and function for up to a year; however, LR-PRP was associated with a significantly higher risk of local adverse reactions, including increased pain (OR = 1.64, 95% CI: 1.29–2.10, *p* = 0.01) and swelling (OR = 1.56, 95% CI: 1.22–1.99, *p* = 0.02), compared with LP-PRP. From a nursing perspective, LP-PRP may therefore be preferable for intra-articular and dermatologic indications to minimize inflammatory burden, whereas LR-PRP may be selectively applied in tendon or ligament injuries where controlled inflammation supports tissue remodeling (Kim et al., 2021). Accordingly, accurate recognition, documentation, and differentiation between expected inflammatory responses and pathological complications are essential nursing responsibilities to ensure patient safety and maintain therapeutic trust.

Patient education and expectation management represent another critical yet underdeveloped aspect of PRP implementation. Many patients initially perceive PRP as a rapid or definitive cure, often due to insufficient pre-treatment counseling. Evidence suggests that unrealistic expectations may contribute to dissatisfaction, premature discontinuation, or poor adherence to follow-up care (Fitzpatrick et al., 2017). Nurses play a central role in addressing this gap through structured, multimodal education strategies, including verbal counseling, written materials, anatomical explanations, and audiovisual tools. Psychological responses, including procedural anxiety or frustration during delayed recovery, should be actively assessed, with validated tools such as the State–Trait Anxiety Inventory facilitating early identification and intervention.

The lack of individualized, nursing-driven PRP strategies further limits effective implementation. Current protocols are predominantly physician- or device-determined and may insufficiently account for nursing workload, patient frailty, or socioeconomic constraints. For example, LP-PRP may reduce inflammatory burden in older adults or individuals with chronic inflammatory conditions, whereas injection-based PRP protocols for DFUs require more intensive nursing monitoring compared with topical formulations, despite offering superior tissue penetration and early wound closure (Saad Setta et al., 2011). Consideration of patient mobility, health literacy, home support, and follow-up capacity is therefore essential.

Looking ahead, the expansion of nurse-led PRP care will require greater emphasis on nursing-specific evidence generation, digital monitoring, and competency development. To date, most PRP studies prioritize clinician-centered endpoints, while outcomes central to nursing practice—such as longitudinal healing trajectories, functional recovery, pain control, and patient satisfaction—remain underreported (Martinez-Zapata et al., 2016). Emerging technologies, including mobile health platforms, wearable wound sensors, artificial intelligence-assisted imaging, and predictive analytics, offer promising tools to support nursing decision-making, triage, documentation, and remote follow-up (Chen et al., 2024; Stefanelli et al., 2025). Integrating these innovations into electronic medical records and telehealth systems may strengthen continuity of care and patient self-management, positioning PRP as a model for digitally enabled, nurse-led regenerative therapy.

Investment in standardized education programs, certification pathways, and simulation-based training is essential to ensure competency, reduce inter-operator variability, and strengthen nursing leadership in regenerative medicine. Within an evolving healthcare landscape characterized by biologic, personalized, and multidisciplinary care, PRP therapy represents a meaningful convergence of nursing science, clinical innovation, and patient-centered practice.

## 6 Conclusion

PRP therapy represents a biologically grounded and clinically versatile intervention with increasing relevance to contemporary nursing practice. Across musculoskeletal care, wound management, rehabilitation, and aesthetic nursing, PRP has demonstrated potential to support tissue regeneration and functional recovery when integrated into structured, nurse-led care pathways. This review highlights the biological mechanisms underpinning PRP activity alongside practical nursing considerations throughout the treatment continuum. As PRP adoption continues to expand, the development of standardized nursing competencies, formulation-aware clinical decision-making, and evidence-informed protocols will be essential to optimizing patient outcomes. Multidisciplinary collaboration and nursing leadership remain central to translating PRP from a technical procedure into holistic, patient-centered regenerative care.

This review has several limitations. The included studies demonstrate considerable heterogeneity in PRP preparation techniques, formulations, dosing regimens, and outcome measures, limiting direct comparison and robust evidence synthesis. Moreover, many clinical studies prioritize physician-centered or procedural endpoints, with limited attention to nursing-specific outcomes. These gaps highlight the need for higher-quality, nurse-led research employing standardized protocols and clinically meaningful nursing metrics.

## References

[B1] Ahramiyanpour N. Kaveh R. Lotfi E. Rastaghi F. (2025). Optimizing post-acne scar treatment: a pilot comparative study of endo-radiofrequency subcision with and without platelet-rich plasma. J. Cosmet. Dermatol. 24, e70345. 10.1111/jocd.70345 40665746 PMC12264461

[B2] Alam M. Hughart R. Champlain A. Geisler A. Paghdal K. Whiting D. (2018). Effect of platelet-rich plasma injection for rejuvenation of photoaged facial skin: a randomized clinical trial. JAMA Dermatol 154, 1447–1452. 10.1001/jamadermatol.2018.3977 30419125 PMC6583756

[B3] Asubiaro J. Avajah F. (2024). Platelet-rich plasma in aesthetic dermatology: current evidence and future directions. Cureus 16, e66734. 10.7759/cureus.66734 39268288 PMC11391108

[B4] Bazezew A. M. Getahun Y. Demlie T. A. Ayele D. G. Siyoum T. M. Gedefaw G. D. (2024). Knowledge and associated factors with respect to prevention of post-traumatic compartment syndrome among surgical unit nurses; a multi-center cross-sectional study. BMC Nurs. 23, 164. 10.1186/s12912-024-01806-2 38448942 PMC10916007

[B5] Bilkis F. Shoma F. K. Rahman M. M. Fuad S. M. Mohajan K. Haque F. (2025). Outcome of autologous platelet-rich plasma injection in patients with lateral epicondylitis. J. Sylhet Women’s Med. Coll. 15, 17–24. 10.47648/jswmc2025v15-02-120

[B6] Brinks A. Desai D. D. Needle C. Kearney C. A. Nohria A. Sikora M. (2025). Evaluating the accuracy of patient-reported hair outcomes *versus* trichometric measurements in PRP therapy. Arch. Derm. Res. 317, 772. 10.1007/s00403-025-04264-1 40392319

[B7] Bucak Ö. F. Kalaoglu E. Atasoy M. Coskun E. (2025). Comparative effectiveness of platelet-rich plasma and steroid injections in chronic plantar fasciitis: evaluating pain relief and functional recovery. Foot Ankle Int. 46, 10711007251346784–10711007251346995. 10.1177/10711007251346784 40581848

[B8] Cenni E. Ciapetti G. Granchi D. Fotia C. Perut F. Giunti A. (2009). Endothelial cells incubated with platelet-rich plasma express PDGF-B and ICAM-1 and induce bone marrow stromal cell migration. J. Orthop. Res. 27, 1493–1498. 10.1002/jor.20896 19396860

[B9] Chahla J. Cinque M. E. Piuzzi N. S. Mannava S. Geeslin A. G. Murray I. R. (2017). A call for standardization in platelet-rich plasma preparation protocols and composition reporting: a systematic review of the clinical orthopaedic literature. J. Bone Jt. Surg. Am. 99, 1769–1779. 10.2106/JBJS.16.01374 29040132

[B10] Charles S. Guyotat D. Fontana P. Tardy B. Lecompte T. Chalayer E. (2022). External validation of the MidiCAT variant of thrombography: Comparison with calibrated automated thrombography and study of the centrifugation scheme. Front. Cardiovasc. Med. 9, 998687. 10.3389/fcvm.2022.998687 36337867 PMC9635262

[B11] Chen X. Jones I. A. Togashi R. Park C. Vangsness C. T. Jr (2020). Use of platelet-rich plasma for the improvement of pain and function in rotator cuff tears: a systematic review and meta-analysis with bias assessment. Am. J. Sports Med. 48, 2028–2041. 10.1177/0363546519881423 31743037 PMC7234896

[B12] Chen M.-Y. Cao M.-Q. Xu T.-Y. (2024). Progress in the application of artificial intelligence in skin wound assessment and prediction of healing time. Am. J. Transl. Res. 16, 2765–2776. 10.62347/MYHE3488 39114681 PMC11301465

[B13] Cullen B. Gefen A. (2023). The biological and physiological impact of the performance of wound dressings. Int. Wound J. 20, 1292–1303. 10.1111/iwj.13960 36110054 PMC10031231

[B14] De Pasquale V. Sarogni P. Pistorio V. Cerulo G. Paladino S. Pavone L. M. (2018). Targeting heparan sulfate proteoglycans as a novel therapeutic strategy for mucopolysaccharidoses. Mol. Ther. Methods Clin. Dev. 10, 8–16. 10.1016/j.omtm.2018.05.002 29942826 PMC6011039

[B15] Del Amo C. Perez-Valle A. Perez-Zabala E. Perez-Del-Pecho K. Larrazabal A. Basterretxea A. (2020). Wound dressing selection is critical to enhance platelet-rich fibrin activities in wound care. Int. J. Mol. Sci. 21, 624. 10.3390/ijms21020624 31963580 PMC7013388

[B16] Demyashkin G. Vadyukhin M. Murtazalieva Z. Pugacheva E. Schekin V. Bimurzaeva M. (2024). Novel molecular mechanisms underlying the ameliorative effect of platelet-rich plasma against electron radiation-induced premature ovarian failure. Int. J. Mol. Sci. 25, 10115. 10.3390/ijms251810115 39337598 PMC11432445

[B17] Dhurat R. Sukesh M. (2014). Principles and methods of preparation of platelet-rich plasma: a review and author's perspective. J. Cutan. Aesthet. Surg. 7, 189–197. 10.4103/0974-2077.150734 25722595 PMC4338460

[B18] Dohan Ehrenfest D. M. Bielecki T. Mishra A. Borzini P. Inchingolo F. Sammartino G. (2012). In search of a consensus terminology in the field of platelet concentrates for surgical use: platelet-rich plasma (PRP), platelet-rich fibrin (PRF), fibrin gel polymerization and leukocytes. Curr. Pharm. Biotechnol. 13, 1131–1137. 10.2174/138920112800624328 21740379

[B19] Evans J. P. Maffulli N. Smith C. Watts A. Valderas J. Goodwin V. (2021). Even experts cannot agree on the optimal use of platelet-rich plasma in lateral elbow tendinopathy: an international Delphi study. J. Orthop. Traumatol. 22, 47. 10.1186/s10195-021-00608-5 34825302 PMC8617097

[B20] Everts P. Onishi K. Jayaram P. Lana J. F. Mautner K. (2020). Platelet-rich plasma: new performance understandings and therapeutic considerations in 2020. Int. J. Mol. Sci. 21. 10.3390/ijms21207794 33096812 PMC7589810

[B21] Eymard F. Ornetti P. Maillet J. Noel E. Adam P. Legre-Boyer V. (2021). Intra-articular injections of platelet-rich plasma in symptomatic knee osteoarthritis: a consensus statement from French-speaking experts. Knee Surg. Sports Traumatol. Arthrosc. 29, 3195–3210. 10.1007/s00167-020-06102-5 32583023 PMC8458198

[B22] Filova E. Blanquer A. Knitlova J. Plencner M. Jencova V. Koprivova B. (2021). The effect of the controlled release of platelet lysate from PVA nanomats on keratinocytes, endothelial cells and fibroblasts. Nanomater. (Basel) 11, 995. 10.3390/nano11040995 33924537 PMC8070234

[B23] Fitzpatrick J. Bulsara M. Zheng M. H. (2017). The effectiveness of platelet-rich plasma in the treatment of tendinopathy: a meta-analysis of randomized controlled clinical trials. Am. J. Sports Med. 45, 226–233. 10.1177/0363546516643716 27268111

[B24] Fundiur V. D. Grodetskyi V. K. Yakobchuk S. O. Khomko O. Y. Kozlovska I. M. Fundiur Y. V. (2021). Peculiarities of performing organ-saving amputation of the foot combined with ozone therapy, local application of autologous platelet-rich plasma and vacuum sanation of postoperative wound in patients with ischemic-gangrenous form of diabetic foot syndrom. Clin. Exp. Pathol. 20. 10.24061/1727-4338.xx.2.76.2021.13

[B25] Hall M. P. Band P. A. Meislin R. J. Jazrawi L. M. Cardone D. A. (2009). Platelet-rich plasma: current concepts and application in sports medicine. J. Am. Acad. Orthop. Surg. 17, 602–608. 10.5435/00124635-200910000-00002 19794217

[B26] Hassan S. A. Saade D. S. Kurban M. Rahal J. A. Alameddine R. M. (2025). Evaluating the efficacy of combined platelet-rich plasma and microneedling for aesthetic rejuvenation of the periorbital area: a randomized, blinded cohort study. J. Cosmet. Dermatol 24, e16717. 10.1111/jocd.16717 39645648 PMC11845936

[B27] Hegab A. F. Ali H. E. Elmasry M. Khallaf M. G. (2015). Platelet-rich plasma injection as an effective treatment for temporomandibular joint osteoarthritis. J. Oral Maxillofac. Surg. 73, 1706–1713. 10.1016/j.joms.2015.03.045 25882438

[B28] Heydari P. Zargar Kharazi A. Shariati L. (2024). Enhanced wound regeneration by PGS/PLA fiber dressing containing platelet-rich plasma: an *in vitro* study. Sci. Rep. 14, 12019. 10.1038/s41598-024-62855-w 38797743 PMC11128439

[B29] Hoefer T. Armstrong P. C. Finsterbusch M. Chan M. V. Kirkby N. S. Warner T. D. (2015). Drug-free platelets can act as seeds for aggregate formation during antiplatelet therapy. Arterioscler. Thromb. Vasc. Biol. 35, 2122–2133. 10.1161/ATVBAHA.115.306219 26272940 PMC4587545

[B30] Hu W.-H. Zhang X.-Y. Leung K.-W. Duan R. Dong T.-X. T. Qin Q.-W. (2022). Resveratrol, an inhibitor binding to VEGF, restores the pathology of abnormal angiogenesis in retinopathy of prematurity (ROP) in mice: application by intravitreal and topical instillation. Int. J. Mol. Sci. 23, 6455. 10.3390/ijms23126455 35742898 PMC9223486

[B31] Huang G. Zhang J. Wei Z. Mai Y. Guo J. Jiang L. (2022). Ultrasound-guided injection of autologous platelet-rich plasma for refractory lateral epicondylitis of humerus: case series. J. Pers. Med. 13, 66. 10.3390/jpm13010066 36675727 PMC9860542

[B32] Huang D. Vithran D. T. A. Gong H.-L. Zeng M. Tang Z.-W. Rao Z.-Z. (2023). Effectiveness of platelet-rich plasma in the treatment of achilles tendon disease. World J. Orthop. 14, 485–501. 10.5312/wjo.v14.i6.485 37377997 PMC10292057

[B33] Huang Z. Gu Z. Zeng Y. Zhang D. (2025). Platelet-rich plasma alleviates skin photoaging by activating autophagy and inhibiting inflammasome formation. Naunyn. Schmiedeb. Arch. Pharmacol. 398, 8669–8680. 10.1007/s00210-025-03800-0 39836253

[B34] Hudak P. L. Amadio P. C. Bombardier C. (1996). Development of an upper extremity outcome measure: the DASH (disabilities of the arm, shoulder and hand) [corrected]. The upper extremity collaborative group (UECG). Am. J. Ind. Med. 29, 602–608. 10.1002/(SICI)1097-0274(199606)29:6<602::AID-AJIM4>3.0.CO;2-L 8773720

[B35] Huskisson E. C. (1974). Measurement of pain. Lancet 304, 1127–1131. 10.1016/s0140-6736(74)90884-8 4139420

[B36] Jayaram P. Mitchell P. J. T. Shybut T. B. Moseley B. J. Lee B. (2023). Leukocyte-rich platelet-rich plasma is predominantly anti-inflammatory compared with leukocyte-poor platelet-rich plasma in patients with mild-moderate knee osteoarthritis: a prospective, descriptive laboratory study. Am. J. Sports Med. 51, 2133–2140. 10.1177/03635465231170394 37199381

[B37] Jiang N. Du P. Qu W. Li L. Liu Z. Zhu S. (2016). The synergistic effect of TiO2 nanoporous modification and platelet-rich plasma treatment on titanium-implant stability in ovariectomized rats. Int. J. Nanomedicine 11, 4719–4733. 10.2147/IJN.S113375 27695328 PMC5033614

[B38] Jin K. Pandey N. B. Popel A. S. (2018). Simultaneous blockade of IL-6 and CCL5 signaling for synergistic inhibition of triple-negative breast cancer growth and metastasis. Breast Cancer Res. 20, 54. 10.1186/s13058-018-0981-3 29898755 PMC6000947

[B39] Kanno E. Tanno H. Masaki A. Sasaki A. Sato N. Goto M. (2019). Defect of interferon γ leads to impaired wound healing through prolonged neutrophilic inflammatory response and enhanced MMP-2 activation. Int. J. Mol. Sci. 20, 5657. 10.3390/ijms20225657 31726690 PMC6888635

[B40] Kao D. S. Zhang S. W. Vap A. R. (2022). A systematic review on the effect of common medications on platelet count and function: which medications should be stopped before getting a platelet-rich plasma injection? Orthop. J. Sports Med. 10, 23259671221088820. 10.1177/23259671221088820 35434168 PMC9008823

[B41] Karam F. Sayadi M. Dadi S. Sarab G. A. (2025). Overexpression of miR-192 in fibroblasts accelerates wound healing in diabetic rats: research article. Eur. J. Med. Res. 30, 239. 10.1186/s40001-025-02449-y 40186269 PMC11969854

[B42] Kim J.-H. Park Y.-B. Ha C.-W. Roh Y. J. Park J.-G. (2021). Adverse reactions and clinical outcomes for leukocyte-poor *versus* leukocyte-rich platelet-rich plasma in knee osteoarthritis: a systematic review and meta-analysis. Orthop. J. Sports Med. 9, 23259671211011948. 10.1177/23259671211011948 34277879 PMC8255589

[B43] Kim M. K. Yoon J. A. Yoon S. Y. Park M. Lee W. S. Lyu S. W. (2022). Human platelet-rich plasma facilitates angiogenesis to restore impaired uterine environments with asherman's syndrome for embryo implantation and following pregnancy in mice. Cells 11, 1549. 10.3390/cells11091549 35563855 PMC9101537

[B44] Koç Babayiğit F. Kartal D. Çinar S. L. Borlu M. (2025). Effects of platelet-rich plasma application on hair follicle count, telogen/anagen ratio, and miniaturized hair ratio in patients with androgenic alopecia: alone or in combination with other treatments. J. Dermatol. Treat. 36, 2528343. 10.1080/09546634.2025.2528343 40625221

[B45] Kushida S. Kakudo N. Morimoto N. Hara T. Ogawa T. Mitsui T. (2014). Platelet and growth factor concentrations in activated platelet-rich plasma: a comparison of seven commercial separation systems. J. Artif. Organs 17, 186–192. 10.1007/s10047-014-0761-5 24748436

[B46] Kusuma G. D. Li A. Zhu D. McDonald H. Inocencio I. M. Chambers D. C. (2022). Effect of 2D and 3D culture microenvironments on mesenchymal stem cell-derived extracellular vesicles potencies. Front. Cell Dev. Biol. 10, 819726. 10.3389/fcell.2022.819726 35237601 PMC8882622

[B47] Le A. D. K. Enweze L. DeBaun M. R. Dragoo J. L. (2018). Current clinical recommendations for use of platelet-rich plasma. Curr. Rev. Musculoskelet. Med. 11, 624–634. 10.1007/s12178-018-9527-7 30353479 PMC6220007

[B48] Lee J. Kim D. Jang C. H. Kim G. H. (2022). Highly elastic 3D-printed gelatin/HA/placental-extract scaffolds for bone tissue engineering. Theranostics 12, 4051–4066. 10.7150/thno.73146 35673575 PMC9169369

[B49] Lei X. Cheng L. Yang Y. Pang M. Dong Y. Zhu X. (2021). Co-administration of platelet-rich plasma and small intestinal submucosa is more beneficial than their individual use in promoting acute skin wound healing. Burns Trauma 9, tkab033. 10.1093/burnst/tkab033 35464804 PMC8634292

[B50] Liu T. Ma W. Xu H. Huang M. Zhang D. He Z. (2018a). PDGF-Mediated mesenchymal transformation renders endothelial resistance to anti-VEGF treatment in glioblastoma. Nat. Commun. 9, 3439. 10.1038/s41467-018-05982-z 30150753 PMC6110798

[B51] Liu G.-X. Ma S. Li Y. Yu Y. Zhou Y.-X. Lu Y.-D. (2018b). Hsa-let-7c controls the committed differentiation of IGF-1-treated mesenchymal stem cells derived from dental pulps by targeting IGF-1R *via* the MAPK pathways. Exp. Mol. Med. 50, 1–14. 10.1038/s12276-018-0048-7 29650947 PMC5938007

[B52] Manosalva C. Alarcón P. González K. Soto J. Igor K. Peña F. (2020). Free fatty acid receptor 1 signaling contributes to migration, MMP-9 activity, and expression of IL-8 induced by linoleic acid in HaCaT cells. Front. Pharmacol. 11, 595. 10.3389/fphar.2020.00595 32431615 PMC7216565

[B53] Marck R. E. Gardien K. L. M. Vlig M. Breederveld R. S. Middelkoop E. (2019). Growth factor quantification of platelet-rich plasma in burn patients compared to matched healthy volunteers. Int. J. Mol. Sci. 20, 288. 10.3390/ijms20020288 30642068 PMC6358744

[B54] Marques R. Lopes M. Ramos P. Neves-Amado J. Alves P. (2023). Prognostic factors for delayed healing of complex wounds in adults: a scoping review. Int. Wound J. 20, 2869–2886. 10.1111/iwj.14128 36916415 PMC10410354

[B55] Martinez-Zapata M. J. Martí-Carvajal A. J. Solà I. Expósito J. A. Bolíbar I. Rodríguez L. (2016). Autologous platelet-rich plasma for treating chronic wounds. Cochrane Database Syst. Rev. 2016, CD006899. 10.1002/14651858.CD006899.pub3 23076929

[B56] Matsumura S. Quispe-Salcedo A. Schiller C. M. Shin J. S. Locke B. M. Yakar S. (2017). IGF-1 mediates EphrinB1 activation in regulating tertiary dentin formation. J. Dent. Res. 96, 1153–1161. 10.1177/0022034517708572 28489485 PMC5582682

[B57] Mishra A. K. Skrepnik N. V. Edwards S. G. Jones G. L. Sampson S. Vermillion D. A. (2014). Efficacy of platelet-rich plasma for chronic tennis elbow: a double-blind, prospective, multicenter, randomized controlled trial of 230 patients. Am. J. Sports Med. 42, 463–471. 10.1177/0363546513494359 23825183

[B58] Mogoi V. Elder B. Hayes K. Huhman D. (2019). Effectiveness of platelet-rich plasma in the management of knee osteoarthritis in a rural clinic. Orthop. Nurs. 38, 193–198. 10.1097/NOR.0000000000000556 31124870

[B59] Mohammadi M. H. Molavi B. Mohammadi S. Nikbakht M. Mohammadi A. M. Mostafaei S. (2017). Evaluation of wound healing in diabetic foot ulcer using platelet-rich plasma gel: a single-arm clinical trial. Transfus. Apher. Sci. 56, 160–164. 10.1016/j.transci.2016.10.020 27839965

[B60] Mohammed H. T. Fraser R. D. J. Cassata A. (2025). Impact of digital wound care solution on healing time: a descriptive study in home health settings. PLOS Digit. Health 4, e0000855. 10.1371/journal.pdig.0000855 40445955 PMC12124507

[B61] Moneib H. A. Youssef S. S. Aly D. G. Rizk M. A. Abdelhakeem Y. I. (2018). Autologous platelet-rich plasma *versus* conventional therapy for the treatment of chronic venous leg ulcers: a comparative study. J. Cosmet. Dermatol. 17, 495–501. 10.1111/jocd.12401 28834103

[B62] Montañez-Heredia E. Irízar S. Huertas P. J. Otero E. Del Valle M. Prat I. (2016). Intra-articular injections of platelet-rich plasma *versus* hyaluronic acid in the treatment of osteoarthritic knee pain: a randomized clinical trial in the context of the Spanish national health care system. Int. J. Mol. Sci. 17, 1064. 10.3390/ijms17071064 27384560 PMC4964440

[B63] Moshiri A. Oryan A. Meimandi-Parizi A. (2015). Synthesis, development, characterization and effectiveness of bovine pure platelet gel-collagen-polydioxanone bioactive graft on tendon healing. J. Cell. Mol. Med. 19, 1308–1332. 10.1111/jcmm.12511 25702535 PMC4459846

[B64] Muiños-López E. Delgado D. Sánchez P. Paiva B. Anitua E. Fiz N. (2016). Modulation of synovial fluid-derived mesenchymal stem cells by intra-articular and intraosseous platelet rich plasma administration. Stem Cells Int. 2016, 1247950. 10.1155/2016/1247950 27818688 PMC5080490

[B65] Ni X. Shan X. Xu L. Yu W. Zhang M. Lei C. (2021). Adipose-derived stem cells combined with platelet-rich plasma enhance wound healing in a rat model of full-thickness skin defects. Stem Cell Res. Ther. 12, 226. 10.1186/s13287-021-02257-1 33823915 PMC8022317

[B66] Nilsson P. H. Engberg A. E. Bäck J. Faxälv L. Lindahl T. L. Nilsson B. (2010). The creation of an antithrombotic surface by apyrase immobilization. Biomaterials 31, 4484–4491. 10.1016/j.biomaterials.2010.02.036 20211488 PMC2852686

[B67] O'Connor J. P. B. Carano R. A. D. Clamp A. R. Ross J. Ho C. C. K. Jackson A. (2009). Quantifying antivascular effects of monoclonal antibodies to vascular endothelial growth factor: insights from imaging. Clin. Cancer Res. 15, 6674–6682. 10.1158/1078-0432.CCR-09-0731 19861458 PMC4688942

[B68] Ostapoff K. T. Cenik B. K. Wang M. Ye R. Xu X. Nugent D. (2014). Neutralizing murine TGFβR2 promotes a differentiated tumor cell phenotype and inhibits pancreatic cancer metastasis. Cancer Res. 74, 4996–5007. 10.1158/0008-5472.CAN-13-1807 25060520 PMC4167492

[B69] Pan D. Yang F. Zhu S. Li Y. Ning G. Feng S. (2021). Inhibition of TGF-β repairs spinal cord injury by attenuating EphrinB2 expressing through inducing miR-484 from fibroblast. Cell Death Discov. 7, 319. 10.1038/s41420-021-00705-8 34711831 PMC8553751

[B70] Panova-Noeva M. Wagner B. Nagler M. Koeck T. Ten Cate V. Prochaska J. H. (2020). Comprehensive platelet phenotyping supports the role of platelets in the pathogenesis of acute venous thromboembolism - results from clinical observation studies. EBioMedicine 60, 102978. 10.1016/j.ebiom.2020.102978 32920367 PMC7494681

[B71] Patel H. Pundkar A. Shrivastava S. Chandanwale R. Jaiswal A. M. (2023). A comprehensive review on platelet-rich plasma activation: a key player in accelerating skin wound healing. Cureus 15, e48943. 10.7759/cureus.48943 38106716 PMC10725573

[B72] Perez A. G. M. Lana J. F. S. D. Rodrigues A. A. Luzo A. C. M. Belangero W. D. Santana M. H. A. (2014). Relevant aspects of centrifugation step in the preparation of platelet-rich plasma. ISRN Hematol. 2014, 176060. 10.1155/2014/176060 25006472 PMC4005024

[B73] Putri A. C. Soedjana H. Hasibuan L. Sundoro A. Septrina R. Sisca F. (2025). The effect of platelet rich plasma on wound healing in pressure ulcer patient grade III and IV. Int. Wound J. 22, e70458. 10.1111/iwj.70458 40667675 PMC12265013

[B74] Qiu T. Crane J. L. Xie L. Xian L. Xie H. Cao X. (2018). IGF-I induced phosphorylation of PTH receptor enhances osteoblast to osteocyte transition. Bone Res. 6, 5. 10.1038/s41413-017-0002-7 29507819 PMC5827661

[B75] Rizzetto G. Molinelli E. Radi G. Diotallevi F. Cirioni O. Brescini L. (2022). Role of daptomycin in cutaneous wound healing: a narrative review. Antibiot. (Basel) 11, 944. 10.3390/antibiotics11070944 35884198 PMC9311791

[B76] Robertson C. Baggott J. Duncan J. (2020). A quality improvement project to assess and improve the recognition of compartment syndrome by nurses in the orthopedic department. Cureus 12, e11179. 10.7759/cureus.11179 33262915 PMC7689952

[B77] Rui S. Dai L. Zhang X. He M. Xu F. Wu W. (2024). Exosomal miRNA-26b-5p from PRP suppresses NETs by targeting MMP-8 to promote diabetic wound healing. J. Control. Release 372, 221–233. 10.1016/j.jconrel.2024.06.050 38909697

[B78] Saad Setta H. Elshahat A. Elsherbiny K. Massoud K. Safe I. (2011). Platelet-rich plasma *versus* platelet-poor plasma in the management of chronic diabetic foot ulcers: a comparative study. Int. Wound J. 8, 307–312. 10.1111/j.1742-481X.2011.00797.x 21470370 PMC7950579

[B79] Salamanna F. Maglio M. Sartori M. Tschon M. Fini M. (2020). Platelet features and derivatives in osteoporosis: a rational and systematic review on the best evidence. Int. J. Mol. Sci. 21, 1762. 10.3390/ijms21051762 32143494 PMC7084230

[B80] Salini V. Vanni D. Pantalone A. Abate M. (2015). Platelet rich plasma therapy in non-insertional achilles tendinopathy: the efficacy is reduced in 60-years old people compared to young and middle-age individuals. Front. Aging Neurosci. 7, 228. 10.3389/fnagi.2015.00228 26696880 PMC4674567

[B81] Sebbagh P. Hirt-Burri N. Scaletta C. Abdel-Sayed P. Raffoul W. Gremeaux V. (2023). Process optimization and efficacy assessment of standardized PRP for tendinopathies in sports medicine: Retrospective study of clinical files and GMP manufacturing records in a Swiss university hospital. Bioeng. (Basel) 10, 409. 10.3390/bioengineering10040409 37106596 PMC10135571

[B82] Senet P. Bon F.-X. Benbunan M. Bussel A. Traineau R. Calvo F. (2003). Randomized trial and local biological effect of autologous platelets used as adjuvant therapy for chronic venous leg ulcers. J. Vasc. Surg. 38, 1342–1348. 10.1016/s0741-5214(03)00908-x 14681639

[B83] Somasekharan T. Kasoju N. Raju R. Bhatt A. (2020). Formulation and characterization of alginate dialdehyde, gelatin, and platelet-rich plasma-based bioink for bioprinting applications. Bioeng. (Basel) 7, 108. 10.3390/bioengineering7030108 PMC7552778 32916945

[B84] Stefanelli A. Zahia S. Chanel G. Niri R. Pichon S. Probst S. (2025). Developing an AI-powered wound assessment tool: a methodological approach to data collection and model optimization. BMC Med. Inf. Decis. Mak. 25, 297. 10.1186/s12911-025-03144-y 40783534 PMC12335118

[B85] Stotts N. A. Rodeheaver G. T. Thomas D. R. Frantz R. A. Bartolucci A. A. Sussman C. (2001). An instrument to measure healing in pressure ulcers: development and validation of the pressure ulcer scale for healing (PUSH). J. Gerontol. A Biol. Sci. Med. Sci. 56, M795–M799. 10.1093/gerona/56.12.m795 11723157

[B86] Su W.-H. Wang C.-J. Fu H.-C. Sheng C.-M. Tsai C.-C. Cheng J.-H. (2019). Human umbilical cord mesenchymal stem cells extricate bupivacaine-impaired skeletal muscle function *via* mitigating neutrophil-mediated acute inflammation and protecting against fibrosis. Int. J. Mol. Sci. 20, 4312. 10.3390/ijms20174312 31484417 PMC6747081

[B87] Sundman E. A. Cole B. J. Fortier L. A. (2011). Growth factor and catabolic cytokine concentrations are influenced by the cellular composition of platelet-rich plasma. Am. J. Sports Med. 39, 2135–2140. 10.1177/0363546511417792 21846925

[B88] Torres L. Klingberg E. Nurkkala M. Carlsten H. Forsblad-d'Elia H. (2019). Hepatocyte growth factor is a potential biomarker for osteoproliferation and osteoporosis in ankylosing spondylitis. Osteoporos. Int. 30, 441–449. 10.1007/s00198-018-4721-4 30306221 PMC6449322

[B89] Wang H. Qian J. Zhang Y. Xu W. Xiao J. Suo A. (2017). Growth of MCF-7 breast cancer cells and efficacy of anti-angiogenic agents in a hydroxyethyl chitosan/glycidyl methacrylate hydrogel. Cancer Cell Int. 17, 55. 10.1186/s12935-017-0424-8 28515673 PMC5434523

[B90] Wang P. Li H. Hu Y. Peng X. Ye X. Xu D. (2021). Relationship between ultrasound features and Ki-67 labeling index of soft tissue sarcoma. Front. Oncol. 11, 687878. 10.3389/fonc.2021.687878 34262871 PMC8273548

[B91] Wang X. Jiang C. Zhang Y. Chen Z. Fan H. Zhang Y. (2022). The promoting effects of activated olfactory ensheathing cells on angiogenesis after spinal cord injury through the PI3K/Akt pathway. Cell Biosci. 12, 23. 10.1186/s13578-022-00765-y 35246244 PMC8895872

[B92] Wu Y. Dong Y. Chen S. Li Y. (2014). Effect of platelet-rich plasma and bioactive glass powder for the improvement of rotator cuff tendon-to-bone healing in a rabbit model. Int. J. Mol. Sci. 15, 21980–21991. 10.3390/ijms151221980 25464384 PMC4284689

[B93] Wu Q. Luo X. Xiong Y. Liu G. Wang J. Chen X. (2020). Platelet-rich plasma *versus* hyaluronic acid in knee osteoarthritis: a meta-analysis with the consistent ratio of injection. J. Orthop. Surg. 28, 2309499019887660. 10.1177/2309499019887660 31895000

[B94] Xu J. Chen X. Zhang H. Zhang X. Liu R. Li X. (2025). Platelet-rich plasma relieves inflammation and pain by regulating M1/M2 macrophage polarization in knee osteoarthritis rats. Sci. Rep. 15, 12805. 10.1038/s41598-025-97501-6 40229323 PMC11997200

[B95] Yi H. Li R. Li C. (2025). Platelet-rich plasma for the management of burn wound: a meta-analysis. Int. J. Low. Extrem. Wounds, 15347346251359067. 10.1177/15347346251359067 40671584

[B96] Zhou Y. Zhang J. Wu H. Hogan M. V. Wang J. H. C. (2015). The differential effects of leukocyte-containing and pure platelet-rich plasma (PRP) on tendon stem/progenitor cells - implications of PRP application for the clinical treatment of tendon injuries. Stem Cell Res. Ther. 6, 173. 10.1186/s13287-015-0172-4 26373929 PMC4572462

